# Solving the worldwide emergency department crowding problem – what can we learn from an Israeli ED?

**DOI:** 10.1186/s13584-015-0049-0

**Published:** 2015-10-17

**Authors:** Jesse M. Pines, Steven L. Bernstein

**Affiliations:** Departments of Emergency Medicine and Health Policy & Management, The George Washington University, Washington, DC USA; Department of Emergency Medicine, Yale University School of Medicine, New Haven, CT USA; Office for Clinical Practice Innovation, George Washington University, 2100 Pennsylvania Ave., N.W. Room 314, Washington, DC 20037 USA

## Abstract

ED crowding is a prevalent and important issue facing hospitals in Israel and around the world, including North and South America, Europe, Australia, Asia and Africa. ED crowding is associated with poorer quality of care and poorer health outcomes, along with extended waits for care. Crowding is caused by a periodic mismatch between the supply of ED and hospital resources and the demand for patient care. In a recent article in the Israel Journal of Health Policy Research, Bashkin et al. present an Ishikawa diagram describing several factors related to longer length of stay (LOS), and higher levels of ED crowding, including management, process, environmental, human factors, and resource issues. Several solutions exist to reduce ED crowding, which involve addressing several of the issues identified by Bashkin et al. This includes reducing the demand for and variation in care, and better matching the supply of resources to demands in care in real time. However, what is needed to reduce crowding is an institutional imperative from senior leadership, implemented by engaged ED and hospital leadership with multi-disciplinary cross-unit collaboration, sufficient resources to implement effective interventions, access to data, and a sustained commitment over time. This may move the culture of a hospital to facilitate improved flow within and across units and ultimately improve quality and safety over the long-term.

## Background

In a recent Israel Journal of Health Policy Research (IJHPR) article, Bashkin et al. describe factors associated with prolonged length of stay (LOS) within a community emergency department (ED) in Israel [1]. Two non-clinician observers monitored the activities of patients and their ED providers from ED arrival to discharge or admission to a hospital ward. The average length of stay in the sample exceeded seven hours. In the end, the authors present an Ishikawa diagram describing several factors related to longer LOS, including management, process, environmental, human factors, and resource issues.

Prolonged ED LOS and ED crowding are highly prevalent and important issues facing hospitals not only in Israel but also globally, with studies demonstrating frequent crowding in North America (the U.S. and Canada primarily), Europe, Australia, and in parts of Asia and Africa [2]. To understand ED flow, it is important first to define prolonged ED LOS and ED crowding as distinct but related concepts. “Crowding” refers to a congested ED where people experience long waits to be seen, and may have prolonged treatment times. By contrast, “prolonged LOS” – or long treatment times—is one of the causes for ED crowding, along with other issues like high ED volume. ED LOS is a chief determinant of the “Throughput” component of the Input-Throughput-Output conceptual model of ED crowding [3].

Over the past two decades, a broad literature has described the causes, consequences, and solutions to ED crowding and prolonged ED LOS, describing ED crowding as an important quality and safety issue [4–6]. When people come to a crowded ED with their health emergency, they receive poorer quality of care and experience poorer health outcomes, along with having extended waits for care. Patients in a crowded ED experience important delays in care, sometimes for life-saving interventions, higher rates of errors and adverse events, and poorer survival [7].

As for the causes of ED crowding, it comes down to the simple concept: crowding is caused by a periodic mismatch between the supply of ED and hospital resources and the demand for patient care. Care demands include provider evaluation and bedside interventions, or other tasks that providers must complete; for example, administrative tasks to complete care processes or chart documentation.

Care delivery requires several resources, including providers to do the work, a physical space to complete the work, and complex interdependent resources – such as radiology, laboratory, or the inpatient wards, each of which has their own provider, space, and capacities to complete tasks. The ED is dependent on the efficient functioning of these interdependent units to sustain good flow. One of the major causes of crowding is poor availability or supply of inpatient beds: throughout the day, as the number of admitted patients occupies more and more of the ED, the ED has fewer available resources to treat the new undifferentiated patients [8]. ED crowding is “periodic” because most EDs are not crowded all the time. For example, many EDs in the U.S. are crowded at 4–8 PM, which is a peak for ED census and demands for care, while at 4 AM many EDs have lower volume and patients do not experience waits. Put simply, ED crowding occurs when the demand for care exceed resource supplies. EDs are dependent on the efficiency of other interdependent hospital units, and EDs are frequently, but not always crowded.

## The study by Baskin and colleagues

To delve into this complex topic and understand the causes for prolonged LOS, Bashkin and her team spent time in the ED observing factors that led to longer ED LOS. What they found were a variety of elaborate, inefficient processes in the ED and across the hospital, where several factors, many of them interdependent, led to prolonged LOS and ultimately higher levels of ED crowding.

Factors were related both to demand for care and supply of resources. For example, an environmental factor related to ED demand that led to prolonged lengths of stay was described as “patient overload.” Insufficient resources were available to handle the number of presenting patients. In addition, inefficient processes led to delays in care such as patient handovers and the lack of continuity of care across shifts, communication failures, and the lack of important information. ED providers were also required to call multiple services to arrange for hospital admissions, or transferring patients back to the ED for disposition decisions, resulting in higher workload demands for ED providers. Other workload problems also dominated the results, with issues such as “too many forms to fill,” a widespread refrain in highly regulated environments like healthcare. “Frequent interruptions” were also identified which is a common issue in the busy ED environment. There were also problems of limited supply, such as medical devices, which led to prolonged LOS, as well as high occupancy rates on the inpatient services. Specifically, when it came to ED boarding, the authors estimated that nearly 40 % of prolonged LOS for admitted patients was spent boarding in the ED awaiting inpatient beds. However, while the focus of the authors’ observations was to seek out problems, solutions to improve ED flow that directly address identified problems require a broader approach.

## Solutions to improve ED flow

To reduce ED crowding and shorten prolonged ED LOS, there are three basic approaches based on the demand–supply theory: reduce care demands, increase resources, or better match demand and supply. Reducing demand can be achieved by lowering the number of ED visits, eliminating unnecessary steps in ED care and streamlining how patients move through the system. It is notable that many of the factors identified by the authors in this study are potentially amenable through process improvement methodology, such as Lean or Six Sigma, that reduce “wasteful” steps through process redesign [9]. One example of streamlining the front-end is to bring the decision-maker to the patient earlier in the process (i.e. physician-in-triage programs) [10]. In addition, eliminating front-end steps that don’t add value can be effective, such as what is called “immediate bedding” or rapidly placing patients in empty beds as opposed to triage when ED beds are empty [11]. Another important way to reduce demand is to reduce the intensity of care in safe ways, such through the use of clinical decision rules. For example, the Pulmonary Embolism Rule-Out Criteria (PERC) rule is an evidence-based tool that can safely reduce CT use for suspected pulmonary embolism [12]. When the intensity of care is reduced, workload falls and crowding can be reduced. In addition, reducing variation in care through the use of other protocols, such as those related to hospital admission, may be an important way to reduce care demands [13].

Inefficiencies also can be reduced on the back-end, where programs can reduce the practice of boarding admitted patients [14]. Examples of programs aimed at reducing boarding include full-capacity protocols that push ED boarders to inpatient hallways during episodes of high crowding [15], hospital-level interventions that reduce the variability of inpatient bed demands (i.e. surgical schedule smoothing) [16], or other policies intended to improve the use of inpatient beds (i.e. pooling inpatient units).

Directly impacting the supply of resources by recruiting additional staff or by extending the weekly work hours of existing staff can be costly, and may not always result in a return on investment [17]. EDs tend to be built and staffed for the average day, so when a high volume/workload day occurs (sometimes daily ED volume can vary 30 % from day-to-day), there is demand–supply mismatch, patients have to wait for care, and crowding occurs. Matching supply and demand may be a better approach, specifically trying to increase resource supplies (e.g. ED beds and staff) during times of crowding. One example is an ED design that allows for internal, rather than external, waiting areas: in this process, a patient is seen immediately after arrival, and certain patients (e.g. those able to sit in a chair) are placed in an internal waiting area and receive treatment or wait for tests to be done. As opposed to waiting in a pre-evaluation phase, this process, sometimes called “split-flow,” [18] allows the ED to flex up and down more efficiently and get care started earlier. Another supply side intervention is the ability to flex staff up and down similarly, by increasing staffing during days or times when workloads are high.

A final insight is the recognition that ED and hospital culture can also contribute to poor flow. Crowding is a result of staff, space, and processes but it is also a result of how well teams work together within and across units [19]. For example, hospitals with a silo mentality that only consider their own flow and do not recognize crowding as a hospital-wide issue can perpetuate dysfunction in other units while protecting their own staff. The authors describe a process of services refusing to take patients causing the ED to “start over” when patients were deemed inappropriate for their unit. While this “protects” staff on a clinical service, such as orthopedics, this further crowds the ED, leading to longer waits and worse care and outcomes. The ethics of such practices have been discussed elsewhere [20].

## Conclusions

Solutions to ED crowding are complicated, time-consuming, and involve considerable staff time, effort, and investment to change culture and process. To spark and sustain change, an institutional imperative is required, supported by the highest levels of leadership. Because many hospitals have not risen to the challenge to reduce ED crowding [8], several countries have instituted mandates for length of stay such as in England and Australia [21, 22]. In the U.S., ED crowding metrics have been reported as national quality metrics and may in the future be linked to pay-for-performance programs. [23] Despite the impetus, successful approaches require senior and local leaders to bring together multi-disciplinary teams across units to work towards a common goal, and not only identify problems – as the authors did in this study – but also create solutions, to allow multi-disciplinary teams to serve as change agents with resources and data, and sustain improvement over the long term.
